# HaRePo (harm reduction by post): an innovative and effective harm reduction programme for people who use drugs using email, telephone, and post service

**DOI:** 10.1186/s12954-020-00403-1

**Published:** 2020-08-24

**Authors:** Magally Torres-Leguizamon, Emmanuel G. Reynaud, Thomas Néfau, Catherine Duplessy

**Affiliations:** 1 SAFE, 11 avenue de la Porte de la Plaine, 75015 Paris, France; 2 School of Biomolecular and Biomedical Science, University College Dublin, Dublin, Ireland

**Keywords:** Harm reduction programme, Remotely service, HR counselling, HR tool distribution, HR by mail, HR by telephone, HR for hard-to-reach PWUD

## Abstract

**Background:**

Despite multiple harm reduction (HR) programmes worldwide, there are still an important number of people who use drugs (PWUD) who do not access those services. Their difficulties to obtain HR tools are due to their inability to reach such services (remoteness and/or limited customer service hours), costs, quantitative restrictions, fear of judgement, lack of confidentiality in pharmacy, and unfamiliarity with HR programmes. We tested an innovative approach using the power of remote online communication and the national postal distribution network to improve HR tool access and counselling.

**Methods:**

Based on these observations, SAFE association created HaRePo in 2011, a free and confidential programme designed for people who have difficulties accessing HR tools and counselling. PWUD can access the programme by phone and/or email. An HR professional delivers HR counselling and HR tools and connects PWUD to other HR services, medical, and social workers. HR tools are prepared and sent according to the person’s needs through the French postal service to consumers across Metropolitan France and overseas territories.

**Results:**

Since 2011, 1920 PWUD have benefited from HaRePo: 10,450 parcels were sent accounting for more than 1.7 million syringes and 6 million HR-related items. HaRePo receives positive feedback from PWUD who have improved their practices through remote but trusted communication. The percentage of people that, after joining the programme, never reuse and/or share HR tools have significantly increased. On average, 71.5% of beneficiaries never reuse syringes and 81% do not reuse needles. And they are 98.5% consumers who never share syringes and 99% needles any longer. Between 44 and 80% HaRePo beneficiaries have reported that their drug-related practices (injection, inhalation, and snorting) are now safer. Finally, between 39 and 53% HaRePo consumers declared that their overall physical state has improved (e.g. venous condition, the appearance of point of injection, swelling of arms, legs, and hands).

**Conclusion:**

HaRePo is an innovative HR programme efficient for hard-to-reach PWUD. It shows evidence of a positive feedback loop for PWUD in improving their practices. Finally, HaRePo represents a clear benefit for health authorities in France, who decided to expand the programme in 2016.

## Background

Harm reduction (HR) programmes consist in providing support and HR tools to PWUD to reduce the harms associated to drug consumption. They result in significant reduction of health risks for drug consumers. HR is one of the most efficient strategies used in order to decrease human immunodeficiency virus (HIV) and hepatitis transmission [1–3]. HR also reduces other health risks associated with drug use like skin and soft tissue infections, which involve microbial invasion of the skin and underlying soft tissues [4]. HR strategies can also reduce overdose [5]. Moreover, HR provides a gateway to drug treatment programmes for drug consumers and decreases social risks by supplying information and advice in a non-judgmental manner [6].

In France, HR started in 1987 with a Minister of Health decree allowing the free sale of syringes in pharmacy. This scheme was subsequently supplemented by other programmes such as syringe exchange programmes in 1990 and opioid substitution programmes in 1994. Finally, HR was established by law in 2004 [7]. In 2005, the care and support centres in harm reduction for drug consumers called CAARUD (Centres d’Accueil et d’Accompagnement à la Réduction des risques pour Usagers de Drogues in French) were created. Their missions are to welcome, inform, and personalize counselling for PWUD. PWUD support includes the distribution of HR tools, personal care support and access to the general healthcare system, and detection of transmissible diseases; the low threshold structures’ missions are detailed in Decree 2005-1606 of December 19, 2005 [8]. The same legal structure also created the CSAPA care centre for addiction support and prevention (Centre de Soin, d’Accompagnement et de Prévention en Addictologie, in French) whose mission is to provide prevention and care for people who suffer from addictions (e.g. drugs, alcohol gambling, screens, sex). Despite the French governmental actions concerning HR, there are still many PWUD who have difficulties to access to HR services in the country [9]. In fact, a growing number of PWUD have reported difficulties in finding sterile materials. Several reasons have been highlighted: difficulty of accessing HR structures (distance and/or limited customer service hours), material cost and/or lack of confidentiality in pharmacies, material quantity restrictions, unfamiliarity with HR programmes, fear, and shame. These difficulties induce high-risk behaviour in drug consumption (e.g. injection practices), like reuse and sharing of syringes and HR tools. Most non-urban areas have no specialized care infrastructure to diagnose, monitor, and accompany PWUD [10], and indeed historically, drug use and PWUD have been studied and discussed primarily from an urban perspective. Not only have consumers of non-urban areas not been studied or supported, but these areas are hardly supervised by the authorities and are therefore commonly used for drug trafficking [11]. This information is important because the drug trafficking routes are predictive of HIV spread in rural areas, as has been demonstrated in China [12], India [13], and Southeast Asia [14]. Studies about PWUD living in rural areas are scarce. Only a few studies conducted in the Appalachians (USA) [15] or remote Australia [16] attempted to draw a coherent picture of the situation and needs of these populations. Rural populations seem to get their equipment from several sources but do not count on pharmacies [17]. In the USA, recent reports show a sharp increase in the number of new cases of hepatitis C among PWUD living in non-urban areas, especially among young people. In 30 out of 34 states, incidence of hepatitis C has increased between 2006 and 2012, particularly in rural counties east of the Mississippi. The absence of HR centres or syringe exchange practices in rural areas is believed to be one of the causes of the increased incidence of HCV (hepatitis C virus) [18].

Based on these observations, we created HaRePo (Harm Reduction by Post) in 2011, an individualized, customized, confidential, and free HR service with 3 main objectives: (1) to facilitate the access of HR-related items, especially for consumers who do not access and/or have difficulties accessing the classical HR network in sufficient quantity and variety to satisfy their needs; (2) to provide HR counselling and information about health risks associated with drug use and other harmful behaviours (like reuse and sharing of syringes); and (3) to connect consumers with additional legal, healthcare, or other HR services. To our knowledge, HaRePo was the very first programme in the world to propose such kind of services to PWUD. The programme looks to build new HR alternatives for PWUD.

The HaRePo programme is managed by the SAFE association who has developed for the last 25 years several HR strategies in France. SAFE is a pioneer in the creation and evolution of HR paraphernalia: filtration tools [19], injection kits (including an efficient filter against bacteria), inhalation material (proposing free street-based automatic inhalation kit dispensers), and others. SAFE also manages 90 of 300 street-based automatic injection kit dispensers (AIKD) in France. SAFE has contributed to the evaluation of the AIKD as a part of a comprehensive HR strategy [20]. It has also participated in the development of a method using mass spectrometry detection to analyse residual content of used syringes [21, 22] and has provided information for HCV studies [23].

In this study, we present and analyse the results of 7 years of HaRePo activity. This alternative HR programme has established a country-wide and overseas postal-based HR service for hard-to-reach PWUD.

## Methods

### What is HaRePo?

HaRePo (Harm Reduction by Post) is an innovative HR programme designed for people who have difficulties accessing HR tools and advice. HaRePo is a postal delivery service combining regular national postal service with a well-established social and personal communication scheme (professionals respond to telephone calls and emails during in the day) to provide HR counselling and information. Consumers typically receive HR tools 2 days after the initial contact.

### How does HaRePo work?

In 2011, SAFE advertised HaRePo by a short message on their website (http://www.safe.asso.fr) and on the most frequented online forum of drug consumers in France (PsychoACTIF, https://www.psychoactif.org). Since then, the number of consumers has consistently increased, mainly by word of mouth. PWUD can access the programme via a 24/7 telephone line (a voice box records orders outside working hours) and/or by email.

**Fig. 1:**
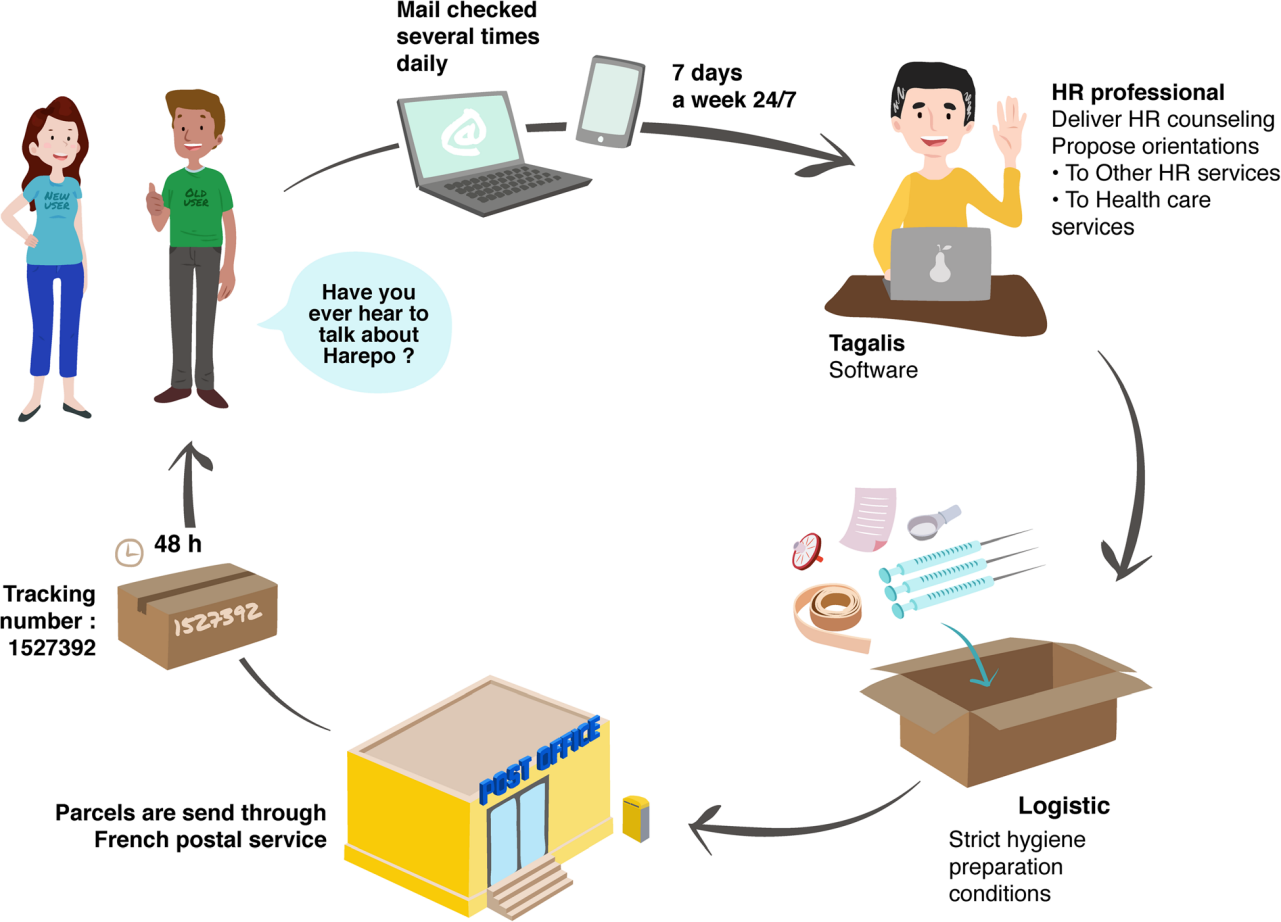
The HaRePo workflow

Professionals are available to answer questions, deliver HR counselling, and connect consumers with other services when necessary (e.g. face-to-face HR services, diverse healthcare services). Then, professionals record orders of HR tools according to the consumer’s needs (e.g. substance consumed, consumption frequency, practices) and tailor the quantity of HR tools sent out. The applicants are identified by a code to prevent recording of personal information. Thus, confidentiality is ensured and strictly follows the guidelines of the National Commission for Informatics and Liberties (CNIL). Once the HR tool order is registered, a logistical team prepares the parcels following strict hygiene preparation conditions. Parcels are finally sent in unmarked packaging through the French postal service to consumers registered across France including French overseas territories. Each parcel has a tracking number to track delivery and delays. Usually, consumers receive parcels 48 h after the initial contact (Fig. 1). The HR tool list provided by the HaRePo programme is presented in an additional file [see Additional file 1].

In order to monitor the entire programme and to ensure consumers’ confidentiality, a software was developed by the company TAGALIS (www.tagalis.com) specifically for this programme. Each consumer is anonymized by encoding a specific ID code. This software allows us to register (i) consumer information (e.g. gender, age, consumer housing, and working situation), (ii) HR item delivery history, (iii) psychoactive substances used, (iv) characteristics and improvement in consumers’ HR practices (e.g. sharing paraphernalia, reusing syringes), and (v) HR advice and references provided. This allows for a secure but accessible data structure for monitoring. So far, all such information has been recorded for 1920 PWUD during the 7 years of the study. The study including questionnaire development, data analysis, and writing was performed by the association SAFE who developed and delivered the HaRePo programme.

### Evaluation of the programme by PWUD

In order to evaluate the programme and its impact on PWUD behaviour, we randomly distributed 300 multiple choice questionnaire (MCQ) with comment boxes from April to June 2018 to HaRePo consumers. Each questionnaire was distributed with a short instruction manual. The only inclusion criterion was for the respondent to be registered in the HaRePo programme since 2017. The questionnaires were sent within the parcels with a pre-stamped envelope. PWUD answered the questionnaire without the presence of any HR professional (questionnaires were answered remotely and anonymously). The response rate was 32%. We analysed and present below results based on 96 answers. It is important to note that our results are based on self-reporting questionnaires and the practices/health improvements were not monitored independently. This is known to induce bias in the responses [24], but some methods exist to reduce such bias [25], and the questionnaire used in this study followed most of those recommendations.

### Data analysis

#### Statistical analysis

All analyses were performed using the software R.3.5.1 (R Core Team, 2018). We use a linear model to study the correlation between two parameters. For instance, we correlate the time since the programme creation and the number of PWUD using the slope of the linear model as a proxy for the adherence rate. We estimated correlation as well as its 95% confidence interval. We then divided our database into four parts, depending on the population density in the consumer living area (with 1 indicating a city and 4 a rural area), and based on the data from the French National Institute for Statistics and Economic Research (INSEE). Currently in France, they represent respectively zone 1 [total 18,219,100], zone 2 [total 24,778,600 habitants], zone 3 [total 19,142,230 habitants], and zone 4 [total 2,533,000 habitants]. We considered that adherence rates for two different zones are significantly different when their respective 95% confidence intervals do not overlap.

We also studied the correlation between PWUD motivations to join the programme and their residence [population density zone as defined above]. We test such correspondence by using Pearson’s chi-square. We considered any difference as significant when the *p* value was ≤ 0.05.

#### Qualitative data

All the qualitative data showed here (mainly testimonies) were collected through email exchanges that were then combined into anonymous reports. They are used only to contextualize the quantitative data. During these exchanges, the discussion is free and the HR professional follows strict rules to avoid judgement and induce trust with the consumer (if it is needed, the HR professional can use motivational interviewing techniques). Thus, consumers can speak freely and share personal feelings. In order to select quotas presented here, we made a keyword research in the reports (chemsex, pharmacy problems, CAARUD/CSAPA problems). Quotas showed here were chosen randomly; thus, we noticed that most of the testimonies are similar and converge into similar topics and opinions.

## Results

### General observations

Since its creation in 2011, almost 2000 PWUD have benefited from the HaRePo programme. PWUD using HaRePo have increased from 42 consumers in 2011 to 881 consumers in 2018 (Fig. S1a). The number of parcels sent increased concomitantly. More than 10,000 parcels have been sent to date, from 71 parcels in 2011 to 3118 parcels in 2018 (Fig. S1b). This accounts for more than 6 million HR-related items (including 1,720,295 syringes). Most of the HR paraphernalia sent corresponds to items used in injection practices: intravenous injection/intramuscular injection/plug [97%] and inhalation/snorting [3%] (Fig. S1c). An important amount of information concerning HR practices was sent within each parcel, including flyers, videos on USB memory stick or CD-rom, and others. The number of HR items distributed by HaRePo has grown steadily from less than forty-five thousand per year in 2011 to more than 1.5 million per year in 2018 (Fig. S1c). The success of the programme is partially due to “word of mouth” communication between consumers benefiting from the programme, in particular through a well-known forum used by PWUD in France called “psychoactive”.

• “Me and my friend saw your add on the Psychoactif website which says that SAFE is setting up a service to access injection equipment by post. We would like to have more information on this service.”

### Consumer profile

#### HaRePo addresses the specific needs of consumers who are not accessing classical HR centres

HaRePo is more accessed by women (25%) than classical HR centres. Women represent 18% of the CAARUD consumer cohort [26]. We did not identify a unique motivation for women to enter the programme, and the following testimonies suggest that motivations might be quite diverse:

- “I am an eternal drug addict. I take subutex. I have the misfortune to shoot it. I appreciated your cream as my arms are now less marked. In addition I can filter the products and I have the HR equipment I need. I am not putting myself in danger. You have no idea how much you are relieving the weight of my guilt. Finally I really take into account that I need to get away from drugs. I am no longer alone facing all this. Thank you.”

- “HaRePo changed my life. I live in a small town and the only pharmacy selling injection kits is 15 km away and it sells them for 3 € each, so I usually use 2 kits for 1 month. Now I can finally do 1 session/1 syringe!”

- “I am afraid of going to the CAARUD alone. I am afraid to be judged. I am a pretty anxious person.”

- “I am a drug addict. It is difficult for me to find HR equipment. I don’t really know the addresses of centres in my town. I do not have money to buy injections kits in pharmacies and I am afraid of people’s gaze at me and my drug problem. I would like to know how much material can I order for a daily consumption of heroin and oxycodone. Among other things, I have heard of “wheel filters” which seems to be very effective in filtering out the excipients of oxycodone but are difficult to find in my situation.”

- “I do not want to go to HR centres because I am afraid to lose my daughter’s custody. I don’t want to expose neither my child nor myself.”

**Fig. 2:**
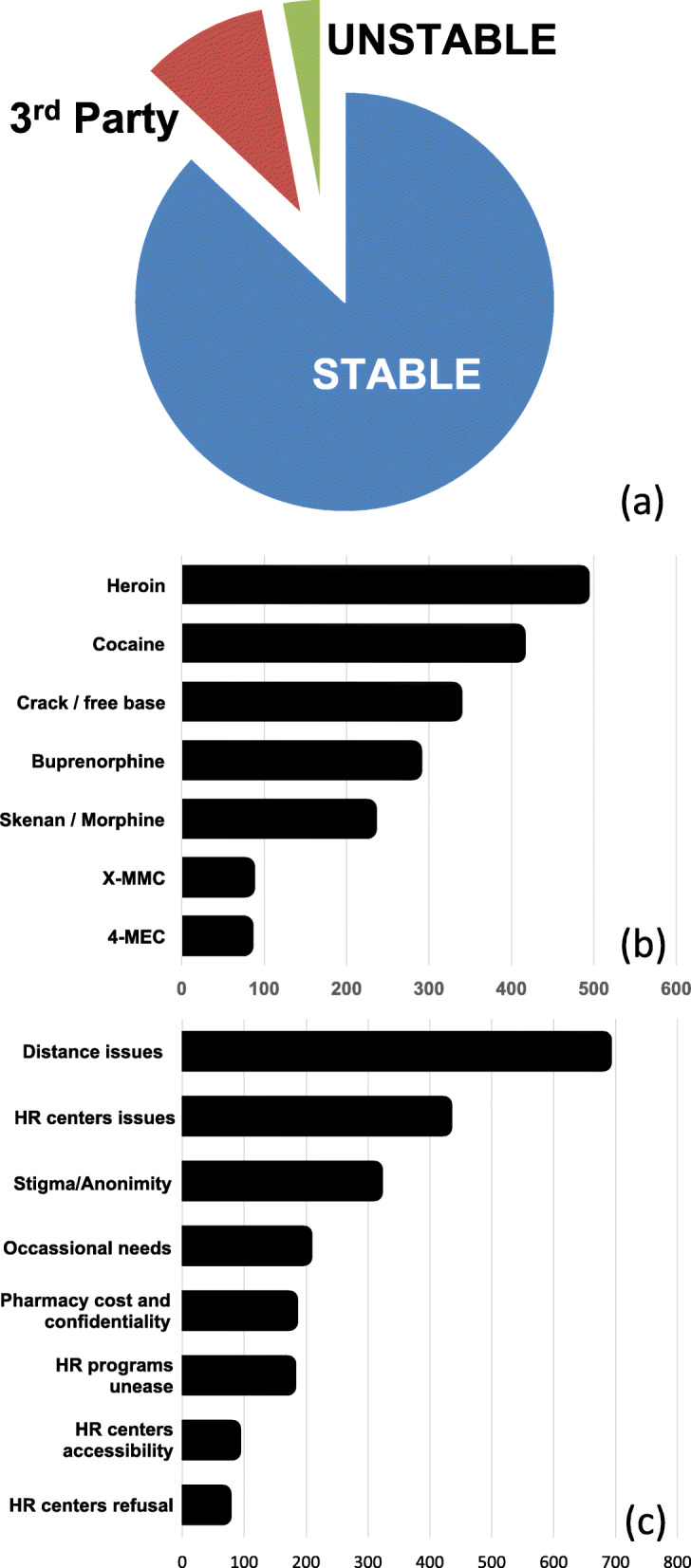
**a** Accommodation of PWUD benefiting from HaRePo. **b** Consumers’ consumption profile. **c** Consumer motivations to join HaRePo

Among programme beneficiaries, the number of people engaging in chemsex (drug use for or during sex) is increased steadily from one in 2012 to 119 in 2018. A total of 170 people engaging in chemsex have benefited from the programme. They represent 9% of the total HaRePo beneficiaries and 11% in 2018. The chemsex practice does not only concern consumers in the urban zones (18% of consumers live in areas of low or very low urban density). HaRePo consumers have a stable accommodation 87% versus 50% in CAARUD [27]. Ten percent of consumers live in accommodations belonging to a third party (i.e. family, friends), and only 3% of consumers do not have a stable accommodation (Fig. 2a).

Most consumers (82%) accessing the programme are injectors, and 94% of them have combined practices: injection and/or inhalation and/or snorting. Most of the people engaging in chemsex declared themselves to be “slamming” (the action of injecting drugs in a sexual context). A small proportion of PWUD do not inject drugs. The structure of our dataset did not allow us to estimate whether there are significant differences depending on their consumption practices. Therefore, we presented here results of both groups (injectors and non-injectors).

The most common substances used are opioids (1035 consumers) like heroin (484 consumers), buprenorphine (280 consumers), Skenan® (255 consumers), and methadone (16 consumers), in addition to oxycodone and Oxycontin®. Moreover, 735 respondents are stimulant consumers with 406 of them consuming cocaine and 329 consuming crack/freebase. Finally, 152 consumers declare consuming new psychoactive substances (NSP), primarily 4-methyl-*N*-ethylcathinone (4-MEC) and x-methylmethcathinone (X-MMC) (Fig. 2b). However, most of PWUD in the programme declare to use multiple drugs.

### Consumer motivations

Consumers decide to use the HaRePo programme for different reasons. The first reason declared by 680 consumers is because of the distance between where they live and the nearest HR centre. The majority of those consumers live in small towns where there are no low threshold structures. The second reason mentioned (442 consumers) is difficulties with the local HR centres like non-compatible service hours, or when HR centres do not have materials or only in insufficient quantities. The third reason for consumers (310 consumers) to join HaRePo instead of HR centres is consumers seek for anonymity and fear of stigma. This result was confirmed by consumers’ testimonies:

- “This service is really great, really suitable for people who, like me, want real anonymity.”

- “Very few people around me are aware of my drug use (two “friends”), and even my family does not know anything about my treatment and it is very good like that at the moment because I hardly need to speak about it. I buy my injection kit in pharmacies but would like filters because I reuse the same syringes at least 4 times and there are only two filters per kit ... the cotton I use filters less well and is not sterile. You will tell me that I could go to a centre or AIKD, I do not dare, I am afraid that friends or acquaintances, worse, my colleagues would pass by and see me exchanging my syringes, entering the centre or because they are inside.”

- “It’s very small here!!! And then, small or not, I know few people who accept the idea that one of their “colleagues” or “partners” is addicted!”

We noted that anonymity was particularly often mentioned by consumers practising chemsex:

- “In fact, I practice slam and saw the recrudescence of HCV contamination (I was infected). I do not dare to go to CAARUD because I will be immediately stigmatized, and the automatic injection kit dispenser is always empty.”

- “I need equipment for injecting and snorting. A friend with whom I practice slam and chemsex told me about you. I do not go to an association because few people know that I use drugs. I do so only in sexual practices. I really do not have a lot of tools. I use the syringe of a friend and I do not want to do it again. To snort I use a ticket or something like that. Is it possible to communicate only through the internet because I am too afraid by phone?”

- “My schedule (working hours) does not correspond at all with CAARUD’s customer services hours.”

- “I practice slam and I live in a small village, and I do not have a vehicle”

- “I practice chemsex, I slam since some time with some partners and I use 3-MMC. In fact, I am a little ashamed to go buy injection kits in pharmacy because of their reaction. So I use or scrounge material from my partners, but some grumble a little”

- “I realize that many slammers are in the same situation. Finding injection kits in pharmacies is very complicated and the new people adopting this practice do not know needle syringe exchange structures or do not want to go to such structures”

Several other motivations were mentioned: during holidays where the centres are closed (196 consumers), similar difficulties encountered with pharmacies (173 consumers), lack of information about HR programmes and tools which exist (170 consumers), not able to go to HR centres because of disability for instance (81 consumers), and finally for personal reasons such as shame (65 consumers). Additionally, we observed that consumers sometimes declare more than one reason that incited them to join the programme (Fig. 2c).

- “I myself am a consumer but I have so far found it very difficult to get equipment, not knowing the address of centres in my city, not having the funds to request in pharmacies and knowing the fear of people’s gaze on my consumption.”

### Motivations to join the programme versus consumers’ residential area

Using linear models, we estimated how rapidly PWUD enter the programme and if this entrance rate depends on their residential area (Fig. S2). We observed that on average, 0.06 PWUD per 100,000 habitants enter the programme each year (Table 1).

**Table 1:** Slopes and their associated 95% CI of the linear model fitted between PWUD benefiting from the programme each year and the time since the beginning of the programme

	Slope (PWUD/100,000 habitants)	95% CI
**Zone density 1**	0.08	0.06–0.10
**Zone density 2**	0.05	0.04–0.06
**Zone density 3**	0.05	0.03–0.07
**Zone density 4**	0.07	0.04–0.09
**Total**	0.06	0.05–0.08

Nevertheless, this rate was not significantly different between the four residential zones (density zones). This means that the density zone (urban or rural) does not play an important role in new HaRePo consumers. Nevertheless, using Pearson’s chi-square tests, we found that residential area has an influence on consumer motivations to join HaRePo. We observed that HaRePo consumers living in zone types 1 and 2 (large and intermediate cities) mainly use the programme because they feel they do not fit in with PWUD who frequent classical HR centres (*p* < 0.001) and because they need timely materials (e.g. HR tools) (*p* < 0.05). Moreover, the principal reason for PWUD living in zone types 3 and 4 (semi-rural and rural areas, respectively) to join the programme is their distance from classical HR centres (*p* < 0.0001) (Table 2).

**Table 2:** Residential area influence on motivations of consumers to join HaRePo

Consumer motivation to join the programme	Population density	*p* value
Consumers that do not recognize themselves in the audience of HR centres	Density 1	*p* < 0.0001
Punctual supplement (e.g. HR tools)	Density 2	*p* < 0.05
Consumers are far from HR centres	Density 3	*p* < 0.0001
Consumers are far from HR centres	Density 4	*p* < 0.0001

Results show that HaRePo receives PWUD from urban and rural population, but the reasons to join the programme are different.

### Programme impact

**Fig. 3:**
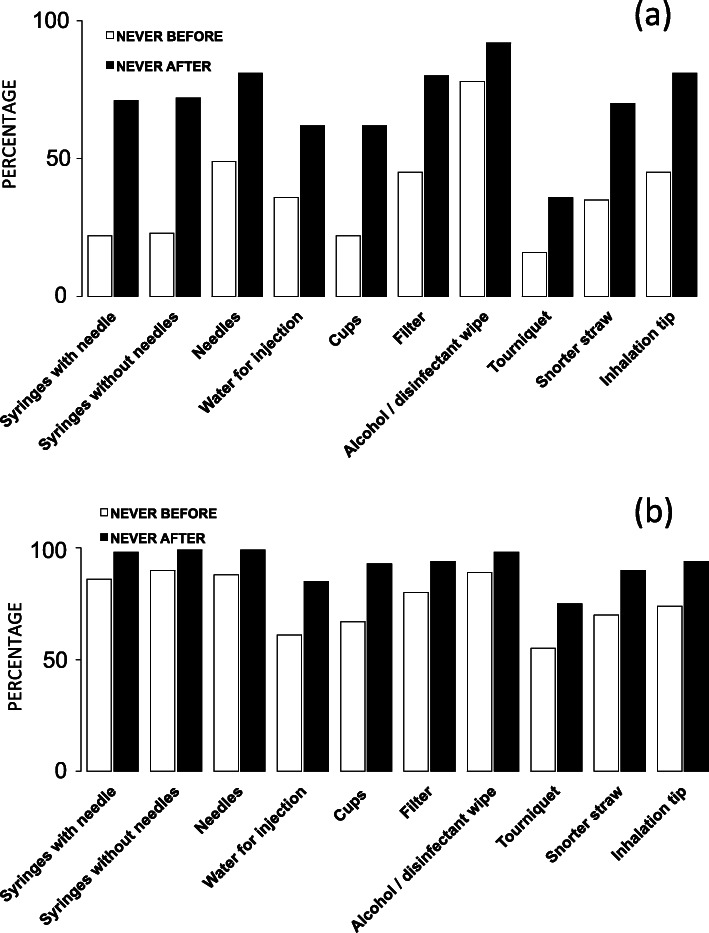
Impact of the programme on tool reuse (**a**) and sharing (**b**)

**Fig. 4:**
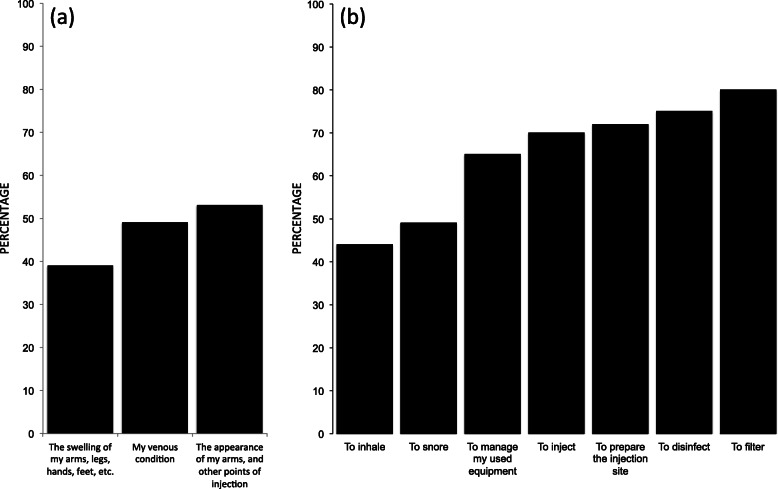
**a** Effect of the programme on PWUD perception of their own health status. **b** Effect of the programme on safety improvement of PWUD consumption practices

The programme seems to induce a positive feedback loop in its consumers. They improve their practices based on remote but trustworthy communication. Furthermore, the percentage of people that, after accessing the programme, never reuse and/or share HR tools have increased. Indeed, 71 to 72% of beneficiaries never reuse syringes and 81% never reuse needles. Concerning other HR items, the level of improvement varies from 14 to 49% (Fig. 3a). Regarding sharing practices, 98 to 99% of consumers declare never sharing syringes and 99% for needles. Moreover, the percentage of consumers that never share other kinds of HR paraphernalia once they have joined HaRePo improved from 9 to 26% (Fig. 3b). In addition, between 39 and 53% of HaRePo consumers declared that the perception of their own health status and physical appearance (e.g. the appearance of injection points, swelling of extremities, veins) has clearly improved (Fig. 4a). Finally, depending on the practices, 44 to 80% of beneficiaries report that since they entered into the programme, the safety of their consumption practices (injection, inhalation, and snorting) has improved or greatly improved (Fig. 4b).

## Discussion

### HaRePo shows evidence of a positive feedback loop as PWUD improve their practices through remote but trusted communication

Establishing remote connections with patients who have difficulty accessing classical health centres is an emerging practice in medicine that has been successful at improving access to health centres in several specific countries such as Australia [28] and South Africa [29] but also seems general to other countries as showed by the review of Win et al. [30]. Remote support groups also help to satisfy the need for emotional support [31]. These benefits extend to the HaRePo programme that has reached populations without easy access to HR structures or tools, including women and/or rural populations. Nevertheless, the benefits of such remote programmes may be limited to patients with relatively high digital literacy or at least with Internet connection [29]. By proposing a phone service, HaRePo overcomes this difficulty and may reach PWUD with low access to Internet facilities. As a whole, HaRePo is a successful programme to improve PWUD practices through remote but trusted communication that helps to have a long-term monitoring of PWUD practices.

### HaRePo: a programme adapted for women

Women are among the priorities of HR strategies in France and in Europe [32], HaRePo seems to reach more women than low threshold structures (CAARUD) in France. A higher proportion of women are found among HaRePo consumers (25%) than PWUD frequenting CAARUD (18%) [26].

Women have been estimated to represent about 40% of consumers in the USA and some parts of Europe, 20% in Eastern Europe, 17% in Central Asia, and 40% in Latin America [33]. Nevertheless, precise quantitative data on women who use drugs are rarely available. Furthermore, the stigma attached to women who use drugs and alcohol has been highly documented; for instance, a survey in Russia by Gorshkova and Shurigina [34] showed that 21% of those interviewed declared that a wife’s drug or alcohol addiction was a valid reason for her husband to beat her. Pinkham and Malinowska-Sempruch [35] examine ways in which gender-related factors can increase female drug consumers’ vulnerability and decrease their access to harm reduction, drug treatment, and sexual and reproductive health services. This study also highlighted the stigma and discrimination for women using drugs during pregnancy and/or with children [36, 37]. In fact, peer pressure encourages women to conceal their drug use, limiting their access to HR information and HR structures [38]. Women who use drugs require new HR approaches to take into account their specific needs about anonymity, confidentiality, sexuality, pregnancy, and motherhood.

### HaRePo: a programme adapted for people engaging in chemsex

The most important reason to use HaRePo services declared by people engaging in chemsex is the fear of stigma as they seek anonymity. The second reason is that HR centres are not compatible with their needs (need specific HR equipment and/or specific quantities, non-compatible service hours). The third reason is that some of them live in small towns far from HR centres. Other reasons often cited are the costs of material and/or the lack of confidentiality in pharmacy and finally the unfamiliarity with classical HR programmes as indicated in consumer testimonies presented in the “Results” section.

A study about chemsex in France [39] pointed out the need to adapt HR equipment delivery for people injecting products in a sexual context. Indeed, their needs are not the same as those of consumers traditionally received in HR structures. Moreover, findings from Bourne et al. [40] indicate that generic drug services, typically designed to address the needs of opiate consumers, may not be sufficiently resourced to address the specific and acute needs of gay men engaged in chemsex. They highlighted the need to facilitate the access to larger quantities of injection equipment for slammers. They noted a persistent difficulty among these groups in reaching structures dedicated to consumers. They also insisted on the importance of designing and promoting HR tools adapted to the products consumed in the context of chemsex [39].

The same study showed either a complete lack of knowledge about HR network and care system, or a great difficulty in mobilizing it for people engaging in chemsex living in rural areas and for those who are outside the gay social network. In fact, these persons prefer to frequent HR services far from their place of residence in order to preserve their confidentiality and/or anonymity. It has been highlighted [41] that a rapidly changing pattern of drug use is emerging (chemsex) that requires health services to find new approaches to HR. Indeed some studies show the greater need of adapted harm reduction services for people engaging in chemsex [42]. In general, authors recommend a non-judgemental and sensitive approach to facilitate conversations about harmful drug use and practices [39, 41]. HaRePo is a HR approach that emphasizes non-judgemental and trustful remote communication. Moreover, we provide HR tools adapted to chemsex context. This may also explain the HaRePo success with people engaging in chemsex.

### Is HaRePo a solution adapted to other countries?

We showed that HaRePo is a successful programme that reaches populations who do not have access to classical HR centres. We identified three main hard-to-reach populations: rural or semi-rural populations, women, and people engaging in chemsex. These populations share the fear of stigma when going to classical harm reduction centres as shown by the consumers’ testimonies presented in the “Results” section.

These populations are not exclusively observed in France, and we consider that our programme is a suitable response to the hard-to-reach populations problematic in other countries with some adaptations regarding local conditions and socio-cultural particularities. The difficulty to access harm reduction tools for these populations was already identified in several countries (see [43] for review). Moreover, several times a year, PWUD in neighbouring countries (especially Belgium, Switzerland, and Spain) contact us to benefit from HaRePo. However, additional financial resources are necessary to be able to send HR tools abroad.

For instance, rural populations suffering from their distance to classical structures were observed in the USA [44], Canada [10], Australia [45], UK [46, 47], China [12, 48], Kenya [49], and Iran [50]. This difficulty due to the distance may in some cases be associated to gender issues [51, 52]. Developing HaRePo-like programme in those countries may partially answer this challenge. The key aspects of HaRePo are anonymity, a rapid adaptation to new types of consumption, and free access to a large diversity of harm reduction equipment adapted to most of the practices we observed. Anonymity is particularly important because the fear of stigma is a limiting factor to take advantage of classical HR centres and to directly access HR equipment in pharmacies [53, 54].

Following these principles, HaRePo may be easily adapted to any other situations respecting some local socio-cultural particularities. Nevertheless, it must be noted that HaRePo benefits from efficient post services which may not be the case in some countries. Moreover, the success of HaRePo is also partially due to the publicity made by PWUD on some self-managed Internet forums where they exchange information on different topics including harm reduction. We regularly present our programme on the main forums, and consequently, countries where PWUD are Internet-connected may be reached more efficiently.

Finally, a similar programme was developed in the USA in 2017 and is called NEXT DISTRO (https://nextdistro.org/). This programme, which developed independently from HaRePo, demonstrates the possibility to develop remote harm reduction policies in different countries as far as they respect the key aspects presented in this study.

## Conclusions

HaRePo is an innovative HR programme efficient for hard-to-reach PWUD including women, people engaging in chemsex, and PWUD living in rural areas. We showed evidence that the programme is efficient to improve PWUD practices through remote but trusted communication using social and digital media. PWUD engaged with HaRePo increase constantly despite it mainly expanding by word-of-mouth. Its success allows us to plan new remote access-related projects: remote screening for hepatitis B and C, HIV by dried blood spot test project, and an online training called naloxone (www.naloxone.fr). We noted that most of the PWUD in the HaRePo programme are people injecting drugs often associated to other consumption practices (inhalation, snort, etc.). A reduced proportion of PWUD do not inject drugs, and the structure of our dataset did not allow us to estimate whether the HaRePo programme has a different impact on injectors versus non-injectors. Since most of the consumers in HaRePo are people injecting drugs, we are confident on the positive effect of HaRePo but further research must be performed to confirm these results in among PWUD who do not inject drugs. Finally, HaRePo represents a clear benefit for the French Health Authority as they decided to expand the programme in 2016. The association SAFE continues to lead HaRePo and proposes different trainings for the new associations (in the different administrative regions of France) that join the programme. However, to ensure the continuity of the programme, more financial support is needed.

## Supplementary information

**Additional file 1:.** HR tools list provided by HaRePo

**Additional file 2: Figure S1.** Numbers of PWUD benefiting from the program per year (a). Numbers of parcels sent per year (b). Number of HR tools sent per year (c). **Figure S2.** Number of PWUD per 100,000 habitants benefiting from the program for different density zones. Black circles stand for all the zones, red triangles for zone 1, green crosses for zone 2, deep blue crosses for zone 3 and light blue squares for zone 4. Lines correspond to linear models fitted for each density zones.

## Data Availability

The datasets analysed during the current study are available at: https://www.dropbox.com/s/q4lwfci2eaqcpdh/Equipment%20distribution%20by%20 year.xlsx?dl=0 and https://www.dropbox.com/s/9ohfaxpjwscnfxg/users%20information.xlsx?dl=0.

## Abbreviations

HaRePo: Harm Reduction by Post
HR: Harm reduction
PWUD: People who use drugs
CAARUD: Centre d’accueil et d’accompagnement à la réduction des risques des usagers de drogues
CSAPA: Centres de soins, d’accompagnement et de prévention en addictologie
CNIL: Commission Nationale de l’informatique et des Libertés
AIKD: Automatic injection kit dispensers
HCV: Hepatitis C virus
NSP: New psychoactive substances
4-MEC: 4-Methyl-*N*-ethylcathinone
X-MMC: x-Methylmethcathinone
