# Supplementary material for: HaRePo (harm reduction by post): an innovative and effective harm reduction programme for people who use drugs using email, telephone, and post service
Source: Harm Reduct J. 2020 Aug 24;17:59. doi: 10.1186/s12954-020-00403-1 (PMC7444046; doi:10.1186/s12954-020-00403-1)
Supplement: Supplementary file 2 — Additional file 2: Figure S1. Numbers of PWUD benefiting from the program per year (a). Numbers of parcels sent per year (b). Number of HR tools sent per year (c). Figure S2. Number of PWUD per 100,000 habitants benefiting from the program for different density zones. Black circles stand for all the zones, red triangles for zone 1, green crosses for zone 2, deep blue crosses for zone 3 and light blue squares for zone 4. Lines correspond to linear models fitted for each density zones. [file 12954_2020_403_MOESM2_ESM.doc]

**Figure S1:** Numbers of PWUD benefiting from the program per year (a). Numbers of parcels sent per year (b). Number of HR tools sent per year (c).

**Figure S2:** Number of PWUD per 100,000 habitants benefiting from the program for different density zones. Black circles stand for all the zones, red triangles for zone 1, green crosses for zone 2, deep blue crosses for zone 3 and light blue squares for zone 4. Lines correspond to linear models fitted for each density zones.
